# A unique amphiphilic triblock copolymer, nontoxic to human blood and potential supramolecular drug delivery system for dexamethasone

**DOI:** 10.1038/s41598-021-00871-w

**Published:** 2021-11-02

**Authors:** Irrum Mushtaq, Zareen Akhter, Muhammad Farooq, Farukh Jabeen, Ashfaq Ur Rehman, Sadia Rehman, Sidra Ayub, Bushra Mirza, Muhammad Siddiq, Farasat Zaman

**Affiliations:** 1 Department of Chemistry, Quaid-I-Azam University, Islamabad, 45320 Pakistan; 2 Department of Chemistry and Biochemistry, Laurentian University, 935 Ramsey Lake Road, Sudbury, ON P3E 2C6 Canada; 3 Department of Pathophysiology, Key Laboratory of Cell Differentiation and Apoptosis of Chinese Ministry of Education, Shanghai Jiao Tong University, School of Medicine, Shanghai, 200025 China; 4 Institute of Biomedical and Genetic Engineering, Islamabad, Pakistan; 5 Department of Biochemistry, Quaid-I-Azam University, Islamabad, 45320 Pakistan; 6 Department of Women’s and Children’s Health, Karolinska Institutet and Pediatric Endocrinology Unit, Karolinska University Hospital, Bioclinicum J9:30, SE-171 74 Solna, Sweden

**Keywords:** Chemical engineering, Materials chemistry, Polymer chemistry

## Abstract

The drug delivery system (DDS) often causes toxicity, triggering undesired cellular injuries. Thus, developing supramolecules used as DDS with tunable self-assembly and nontoxic behavior is highly desired. To address this, we aimed to develop a tunable amphiphilic ABA-type triblock copolymer that is nontoxic to human blood cells but also capable of self-assembling, binding and releasing the clinically used drug dexamethasone. We synthesized an ABA-type amphiphilic triblock copolymer (P2L) by incorporating tetra(aniline) TANI as a hydrophobic and redox active segment along with monomethoxy end-capped polyethylene glycol (mPEG_2k_; M_w_ = 2000 g mol^−1^) as biocompatible, flexible and hydrophilic part. Cell cytotoxicity was measured in whole human blood in vitro and lung cancer cells. Polymer-drug interactions were investigated by UV–Vis spectroscopy and computational analysis. Our synthesized copolymer P2L exhibited tuned self-assembly behavior with and without external stimuli and showed no toxicity in human blood samples. Computational analysis showed that P2L can encapsulate the clinically used drug dexamethasone and that drug uptake or release can also be triggered under oxidation or low pH conditions. In conclusion, copolymer P2L is nontoxic to human blood cells with the potential to carry and release anticancer/anti-inflammatory drug dexamethasone. These findings may open up further investigations into implantable drug delivery systems/devices with precise drug administration and controlled release at specific locations.

## Introduction

Supramolecular biomaterials as drug delivery systems (DDSs) are being designed and investigated for the targeted delivery of therapeutic drugs^1^. Biomaterials possesses a wide range of potential biomedical applications, including but not limited to drug delivery^2^, bone fracture healing^3^, and suture material^4^. Micelles, vesicles, liposomes, nanocapsules, nanospheres, and multifunctional dendritic polymeric systems are examples of supramolecules used as DDSs^5^. An ideal supramolecular biomaterial should be noncytotoxic, biocompatible, biodegradable, and possess good binding efficiency with the entrapped drug^2^. All of these characteristics of supramolecular biomaterials can potentially be achieved through rational design.

Self-assembly is a key approach to form supramolecules through noncovalent interactions^6^. An amphiphilic molecule containing both hydrophobic and hydrophilic segments, when coming close to water, self-assembles into different shapes and sizes of supramolecules depending upon its nature^7–9^. Critical micelle concentration (CMC) is the minimum concentration at which stable micelle formation occurs. The CMC of the self-assembled supramolecule is crucial for the control of their properties^10^. A low CMC value is considered essential for a DDS to avoid premature drug release^11^. Hence various supramolecules with such tunable properties are under the area of interest to improve their use as DDSs. Drug release is an essential aspect of supramolecular DDSs^12^, which can occur via diffusion, degradation, or swelling of the supramolecular core^13^. The trigger of drug release is a critical factor in determining the efficacy of the DDS. Various stimuli such as pH, temperature, and redox potential, etc., are responsible for the drug release^14,15^.

Tetra(aniline) TANI has been utilized in many approaches to overcome the drawbacks associated with the poly(aniline)^16^. In various self-assembled ABA-type triblock copolymers, TANI has been incorporated as a hydrophobic and rigid block along with different hydrophilic blocks^16,17^. Previously, it was shown that thermoresponsive ABA type triblock copolymer (PLGA) possesses great potential application in bone regeneration^18^, and it is an attractive candidate for the controlled release of desired proteins and peptides/molecules^19^.

Triblock copolymers have shown promising results for drug delivery systems while also overcoming the disadvantages of diblock copolymers. A previous study demonstrated that the anticancer drug doxorubicin was highly effective if loaded in triblock copolymers, showing not only higher drug-loading capacity, and faster drug release but also increased cellular toxicity, compared to diblock copolymers^18^. Hydrophobic drugs can easily be incorporated into a DDS consisting of triblock copolymers for both therapeutic and imaging purposes^19^.

In this study, to achieve an improved self-assembling behavior of the synthesized triblock copolymer (P2L), hydrophilicity to the hydrophobic ratio was tuned using a high molecular weight of hydrophilic segments (mPEG_2k,_ M_w_ = 2000 g mol^−1^), compared to our previously reported low molecular weight analog (mPEG_350_, M_w_ = 350 g mol^−1^)^17^. An ideal DDS should possess increased circulation time when administered, and one way to achieve this is to increase the molecular weight of PEG. We also investigated the change in size, morphology and CMC of P2L in the presence and absence of stimuli such as oxidation and pH for potential drug delivery applications. However, the one challenge is to design polymers with minimal toxicity per se to normal cells while carrying a drug of interest, therefore toxicity was also measured in whole human blood samples.

Supramolecular structures than recombinant proteins are not only cost-effective, and possess low risks of cancer/tumor development but can also be tuned as per requirements^20^. Because selective DDS remains the primary hurdle, especially for cancers, systemic or local inflammation, and other diseases, a nontoxic DDS is highly desired. For instance, glucocorticoids (GCs) are anti-inflammatory and anticancer drugs widely used in various conditions. Unfortunately, GCs per se cause insulin resistance and severe side effects in normal tissues, including bones^21,22^. Similarly, cancer drugs such as proteasome inhibitors, cisplatin, and venetoclax designed to target cancer cells also require nontoxic DDSs^23,24^. Thus, a DDS capable of delivering drugs while protecting normal cells, especially whole blood cells, is of great clinical interest.

The present study shows the synthesis of an amphiphilic ABA-type triblock copolymer P2L, self-assembly behavior, and polymer-drug interactions. The novelty of this work resides in computational studies on the binding between P2L and the clinically used drug dexamethasone, drug-uptake and release, and effects on human blood cells and cancer cells in vitro.

## Experimental

### Materials

Monomethoxy-capped polyethylene glycol (mPEG_2k_; M_w_ = 2000 g mol^−1^) was purchased from Sigma Aldrich and azeotropically distilled using dried toluene. Ammonium persulphate (APS), dimethylaminopyridine (DMAP), triethylamine (ET_3_N), and 2,2-diphenyl-1-picrylhydrazyl (DPPH), hydrogen peroxide (H_2_O_2_), double-stranded salmon testes sperm DNA (SS-DNA), tris borate EDTA (TBE) buffer, trichloroacetic acid (TCA), and agarose of analytical grade were purchased from Sigma Aldrich. Trifluoroacetic acid (TFA) dimethylsulfoxide (DMSO), and 4-toluenesulfonyl chloride of analytical grade were purchased from Merk Millipore. Dried tetrahydrofuran (THF), dichloromethane (DCM), ethyl acetate, and diethyl ether were obtained from an Anhydrous Engineering dry solvent system based on Grubbs design^25^.

### Synthesis, deprotection, oxidation, doping, and preparation of micellar solution

#### Synthesis of NH_2_/NH_2_-capped boc-protected tetra(aniline) TANI

We synthesized TANI in three steps as reported previously^17^. First, the synthesis of NO_2_/NO_2_ boc-protected TANI was performed as explained earlier^26^. After that, the reduction of NO_2_/NO_2_ boc-protected TANI was done using ammonium formate and activated Pd on charcoal (10% Pd/C) to achieve the desired product (boc-protected TANI)^27^.

Yield: 91%. FTIR (solid sample, cm^−1^) ν: 3467, 3367 (m, NH stretch), 3041 (w, C–H stretch Ar), 2976, 2931 (s, C–H stretch Al), 1705, (s, ester C=O stretch boc), 1626 (s, NH bend), 1510 (s, C=C stretch Ar), 1155 (s, ester C–O stretch boc), 833 (s, *p*-C_6_H_4_ bend). ^1^H NMR (400 MHz, CDCl_3_): δ ppm 7.12 (m, 8H, Ar–N(boc)–Ar), 6.96 (d, *J* = 8.0 Hz, 4H, Ar–N–boc), 6.63 (d, *J* = 8.0 Hz, 4H, Ar–NH_2_), 4.87 (br. s., 4H, Ar–NH_2_), 1.44 (d, *J* = 4.0 Hz, 27H, boc-*ter*–CH_3_). ^13^C NMR (100.65 MHz, CDCl_3_): δ ppm 152 (ester C=O boc), 139 (NH_2_–Ar), 128, 126, 115 (Ar), 81 (C(CH_3_)_3_ boc), 28 (C(CH_3_)_3_ boc). MS (ESI) m/z: 704.3 [M+Na]^+^.

#### Synthesis of mPEG_2k_-tosylate

The mPEG_2k_ was tosylated as reported previously^28^. In a three-necked round bottom flask, mPEG_2k_ (15.5 mmol, 5.44 g) was dissolved in 5 mL dried THF and the contents were allowed to cool in an ice bath. Next, sodium hydroxide (25 mmol, 1 g, 20% aq. solution) was added to the chilled solution, followed by addition of p‐toluenesulfonyl chloride (14.4 mmol, 2.74 g) in 5 mL of THF using a dropping funnel for 2 h. Thereafter, the solution stirred at 0–5 °C for an additional 2 h was then poured into ice-cold water (20 mL). The extraction of organic fractions was performed with chloroform two times. Next, the combined organic extracts were washed with water, brine (once), and dried over anhydrous MgSO_4_. After that, the solvent was evaporated, which resulted in mPEG_2k_-tosylate as a white solid.

Yield: 90%. FTIR (solid sample, cm^−1^) ν: 2869 (s, C–H stretch Al), 1600, 1456 (s, C=C stretch Ar), 1356, 1178 (s, SO_2_ stretch), 1094 (s, C–O–C PEG). ^1^H NMR (400 MHz, CDCl_3_): δ ppm 7.78 (d, *J* = 8 Hz, 2H, Ar–SO_2_), 7.41 (d, *J* = 8 Hz, 2H, CH_3_–Ar), 4.14 (t, *J* = 8 Hz, 2 H, SO_2_–OCH_2_), 3.53 (m, 176 H, CH_2_–CH_2_–O), 3.36 (s, 3H, OCH_3_), 2.43 (s, 3H, Ar–CH_3_). ^13^C NMR (100.65 MHz, CDCl_3_): δ ppm 141 (H_3_C–Ar), 140 (Ar–SO_2_–O), 130, 128 (Ar), 71 (O–CH_2_–CH_2_), 65 (SO_2_–O–CH_2_), 59 (OCH_3_), 28 (Ar–CH_3_). MS (ESI) m/z: 2189 [M+Na]^+^.

#### Synthesis of amphiphilic ABA-type triblock copolymer coupled via amine linkage (P2)

The synthesized mPEG_2k_-tosylate was coupled with TANI via a nucleophilic substitution reaction^17^. NH_2_/NH_2_ boc-protected TANI (1 eq., 0.73 mmol, 500 mg), mPEG_2k_-tosylate (2.1 eq, 1.53 mmol), and DMAP (52 mg) were transferred to a three-necked round bottom flask and protected under nitrogen atmosphere. After that, DMSO (5 mL) was added, followed by triethylamine (1 mL) and stirred at 65 °C for 24 h. The resulting reaction mixture was cooled and diethyl ether was used for precipitation. Next, the residue was dissolved in THF and reprecipitated using n-hexane. The obtained residue was again dissolved in ethyl acetate and reprecipitated in n-hexane and vacuum dried overnight.

Yield 50%. FTIR (solid sample, cm^−1^) ν: 3366 (s, N–H stretch), 2972, 2874 (s, C–H stretch Al), 1701 (s, ester C=O boc), 1612 (s, N–H bend), 1507 (s, C=C stretch Ar), 1156 (s, C–O–C boc), 1104 (s, C–O–C PEG), 960 (s, C=C bend Ar), 830 (s, *p*-C_6_H_4_ bend). ^1^H NMR (400 MHz, CDCl_3_): δ ppm 7.09 (m, 8H, Ar–N(boc)–Ar), 6.89 (d, *J* = 8 Hz, 4H, NH–Ar–N–boc), 6.53 (d, *J* = 8 Hz, 4H, NH–Ar), 3.6–3.4 (m, 352H, O–CH_2_–CH_2_), 3.3 (s, 6H, OCH_3_), 3.2 (t, *J* = 8 Hz, 4H, NH–CH_2_), 1.35 (s, 27H, boc-*ter*-CH_3_). ^13^C NMR (100.6 MHz, CDCl_3_): δ ppm 159 (ester C = O boc), 137 (Ar–HN–CH_2_), 131–114 (Ar), 81 (C(CH_3_)_3_ boc), 72, 65 (O–CH_2_–CH_2_), 59 (OCH_3_), 46 (HN–CH_2_–CH_2_), 28 (C(CH_3_)_3_ boc). MS (MALDI-TOF) m/z: 4692 [M+Na]^+^.

#### Deprotection of P2 (P2L)

The boc-protected amphiphilic ABA-type triblock copolymer (P2) was deprotected using the reported method^17^. Next, P2 was dissolved in anhydrous DCM and cooled to 0 °C. After this, TFA (equal amount as the solvent) was added and slowly allowed the solution to reach room temperature. The consumption of P2 was monitored using thin layer chromatography (TLC) technique. Then, deprotected solution of P2 was concentrated under reduced pressure using a rotary evaporator. To extract the solution, distilled water, saturated NaHCO_3_ and brine were used. The combined organic fractions were dried using MgSO_4_, filtered, and vacuumed overnight, resulting in a dark grey product.

Yield. 50%. FTIR (solid sample, cm^−1^) ν: 3350 (m, N–H bend), 2917, 2874 (m, C–H stretch Al), 1611 (m, N–H bend), 1501 (s, C=C stretch Ar), 1101 (s, C–O–C PEG), 960 (m, C=C bend Ar), 830 (s, *p*-C_6_H_4_ bend). ^1^H NMR (400 MHz, CDCl_3_): δ ppm 6.8 (m, 8H, Ar–N(boc)–Ar), 6.6 (d, *J* = 8 Hz, 4H, NH–Ar–N–boc), 6.55 (d, *J* = 8 Hz, 4H, NH–Ar), 3.6–3.4 (m, 352H, O–CH_2_–CH_2_), 3.32 (s, 6H, OCH_3_), 3.1 (t, *J* = 8 Hz, 4H, NH–CH_2_), 4.71 (s, NH). ^13^C NMR (100.6 MHz, CDCl_3_): δ ppm 137 (Ar–HN–CH_2_), 129–115 (Ar), 72, 65 (O–CH_2_–CH_2_), 59 (OCH_3_), 45 (HN–CH_2_–CH_2_). MS (MALDI-TOF) m/z: 4392 [M+Na]^+^. GPC (M_W_, M_n,_ PDI: 4172, 3307, 1.2) UV–Vis–NIR (DMSO) λ_max_ = 321 nm (benzenoid π–π*).

#### Oxidation of P2L (P2EB)

P2L was oxidized to its EB state as reported earlier^17^. P2L (1 eq., 0.04 mmol) was dissolved in THF (10 mL) followed by the addition of ammonium persulphate (1 Eq. 0.04 mmol, 9 mg) in 2 M HCl (10 mL) which changed the color of the solution into dark green. Next, the solution was allowed to stir for one hour while adding 2 M NH_4_OH (10 mL). The resulting solution turned blue, which was further stirred for four hours. The reaction mixture was extracted using chloroform (3 × 10 mL), the collected chloroform layers were washed with water (3 × 5 mL) and brine (1 × 5 mL) before drying with MgSO_4_. Finally, the residual solvent was removed, and the product was vacuum-dried overnight.

Yield: 98%. FTIR (solid sample, cm^−1^) ν: 3320 (m, N–H bend), 2917, 2874 (m, C–H stretch Al), 1611 (m, N–H bend), 1600 (s, C=C stretch quinoid ring), 1501 (s, C=C stretch Ar), 1101 (s, C–O–C PEG), 960 (m, C=C bend Ar), 830 (s, *p*-C_6_H_4_ bend). ^1^H NMR (400 MHz, CDCl_3_): δ ppm 6.96 (m, 8H, Ar–N(boc)–Ar), 6.67 (d, *J* = 8 Hz, 4H, NH–Ar–N-boc), 6.53 (d, *J* = 8 Hz, 4H, NH–Ar), 4.8 (s, NH), 3.6–3.4 (m, 352H, O–CH_2_–CH_2_), 3.3 (s, 6H, OCH_3_), 3.1 (t, *J* = 8 Hz, 4H, NH–CH_2_). ^13^C NMR (100.6 MHz, CDCl_3_): δ ppm 137 (Ar–HN–CH_2_), 129–115 (Ar), 72, 65 (O–CH_2_–CH_2_), 59 (OCH_3_), 45 (HN–CH_2_–CH_2_). UV–Vis–NIR (DMSO) λ_max_ = 318 nm (benzenoid π–π*) 578 nm (benzenoid to quinoid).

#### Doping of P2EB (P2ES)

The doping of P2EB was performed using 1 M HCl to obtain the emeraldine salt (ES) state of the TANI segment. P2EB was dissolved in 1 M HCl, stirring overnight until UV–Vis–NIR spectroscopic analysis showed the absence of peak (EB) at 560 nm, indicating the completion of the doping process.

Yield: 98%. UV–Vis–NIR (DMSO) λ_max_ = 318 nm (benzenoid π–π*) 424 nm polaron–π*), 1030 nm (π–polaron).

#### Preparation of micellar solution

Micellar solutions of P2 in LEB, EB, and ES states were prepared by dissolving the pre-determined amount of samples, as explained earlier^29^. For doped and dedoped samples, we used 1 M HCl and 1 M NH_4_OH. The solutions were kept overnight to for self-assembling and then subjected to further studies.

### Characterization of the synthesized triblock copolymers

Perkin Elmer spectrum 100 FTIR spectrophotometer was used to record fourier transform infrared (FTIR) spectra. ^1^H and ^13^C nuclear magnetic resonance (NMR) spectra were detected from VNMRS400 spectrometers using deuterated dimethylsulfoxide-*d6* (DMSO-*d6*) and chloroform (CDCl_3_). Waters 410 GPC instrument was used to record the gel permeation chromatogram using dried THF as an eluent at the flow rate of 1 mL/min at room temperature. MALDI-TOF spectrometry was performed as reported earlier. Applied Biosystems 4700 Proteomics Analyser Matrix-Assisted Laser Desorption/Ionisation (MALDI) spectrometer was used to obtain mass secptra of the products. The solution-state UV–Vis–NIR spectra were recorded on Perkin Elmer Lambda 35 UV–Vis–NIR spectrophotometer. Gamry potentiostat/galvanostat interface 1000 was used to perform cyclic voltammetry using Ag/AgCl and Pt wire as the reference counter electrode respectively in 1.0 M H_2_SO_4_. The working electrode was developed by coating with sample solution on fluorine-doped tin oxide (FTO) glass. A potential range from − 0.1 to 1.0 V was used to measure the cyclic voltammograms at different scan rates of 50–150 mV/s. Critical micelle concentration (CMC) was determined by fluorescence spectrophotometry using Perkin Elmer Precisely LS55 Luminescence spectrometer, using pyrene as a hydrophobic dye. Transmission electron microscopy (TEM) images were collected using a JEOL JEM 1200 EX microscope. Samples were prepared using copper TEM grids coated with carbon film, via the drop-casting method. The particle size distribution was measured utilizing dynamic light scattering (DLS) technique using a Malvern Zeta Sizer Nano series equipped with a laser of wavelength 633 nm. The samples for DLS analysis were prepared in deionized water and filtered through a 0.45 µm nylon filter before the measurements. TESCAN Vega LUM equipment was used to collect the scanning electron microscopy (SEM) images, while drop-casting the miceller solution on the glass surface for SEM assessment.

### Cytotoxicity analysis in the blood (Hemolytic assay) and lung cancer cells

The whole blood samples were collected from three healthy individuals (28 years males) after informed consent and ethical approval from the institutional ethical board at Quaid-i-Azam University. All methods were performed in accordance with the relevant guidelines and regulations*.* The samples were centrifuged at 3000 rpm for 5 min to collect the RBCs pellet. The hemolytic assay was used to measure cytotoxicity as reported previously^30^. Phosphate buffer saline (PBS, 1×, 1L) was prepared by carefully dissolving, 8 g of sodium phosphate, 0.2 g of potassium chloride, 1.44 g of sodium phosphate, and 0.24 g of potassium phosphate in double-distilled water. 1 N hydrochloric acid was used to adjust the pH of the solution to 7.4. The solution was sterilized using an autoclave at 121 °C, for 20 min in its liquid cycle and was stored at room temperature. After washing with 1× PBS (7.4), a 10% suspension of RBCs in PBS was prepared by diluting with 1/10th of its initial volume. The 12.5 µL of each concentration of test triblock copolymers (1–0.06 mg/mL), negative control (PBS), and positive control (1% (v/v) Triton X-100) were mixed with 50 µL of diluted RBCs and 187.5 µL of 1× PBS in different eppendorf tubes. The resulting reaction mixtures were co-incubated at 37 °C for 30 min. Finally, following the centrifugation at 1000 rpm for 1 min, the absorbance of supernatants (100 µL) at 540 nm wavelength was measured on a microplate reader. The percentage of hemolysis was calculated using the following formula^30^.

$$\% {\text{ Hemolysis}} = \left( {{\text{Absorbance of test sample}}/{\text{Absorbance of positive control}}} \right) \times {1}00$$

The human lung cancer cell line NCI-H157 (H157) was cultured in RPMI-1640 medium supplemented with 10% fetal bovine serum and grown at 37 °C in a humidified 5% CO_2_ incubator as reported previously^31^. To investigate effect of P2L alone and P2L + Dexamethasone under low pH conditions on cancer cells proliferation/viability, metabolic viability based high throughput assay using tetrazolium salts like MTT (3-(4,5-Dimethylthiazol 2-yl)-2,5-diphenyltetrazolium bromide) purchased from Sigma-Aldrich was used as reported previously^32^.

### Dexamethasone uptake and release study

Clinically used drug “dexamethasone sodium phosphate” (Venus Pharmaceutical, 4 mg/mL, Batch # DS/0221AF) was used to study whether our synthesized amphiphilic triblock copolymer P2L interact and carry the drug. To investigate the uptake and release of dexamethasone, we used UV–Vis spectroscopy measuring the absorption at 242 nm. The uptake of dexamethasone was estimated by recording the UV–Vis spectra before and after adding the P2L in PBS (pH 7.4). The electrochemical stimulation was performed to study the release of dexamethasone. The amphiphilic triblock copolymer P2L encapsulating the dexamethasone was initially kept under a static oxidation potential of + 0.5 V for 6 min and further incubated for 10 min at room temperature. After applying the electrical stimulation, the release of dexamethasone was measured by recording the UV–Vis spectrum. The passive drug release was also monitored by taking the UV–Vis spectra at predetermined time without applying static oxidation potential.

### Molecular docking studies for polymer–drug binding

The molecular docking approach was applied using computing package by the chemical computing group; Molecular Operating Environment (MOE)^33^. Both the amphiphilic triblock copolymer P2L and drug dexamethasone were protonated, and energy minimized using the default parameters of the MOE, i.e., gradient: 0.05, ForceField: MMFF94X. P2L was selected as a receptor. Further, the protonation was performed using default parameters of the structure preparation module of MOE. Finally, docking was conducted using the default parameters of MOE; Placement: Triangle Matcher, rescoring 1: London dG, Refinement: Forcefield, Rescoring 2: GBVI/WSA. A total of five conformations for the ligand were selected before running the docking protocol. The top-ranked conformations based on docking score (S) were picked for analysis. Drug-polymers interaction was visualized through MOE using an interaction module.

### Statistical analysis

For statistical analysis, Student’s *t* test was applied to compare differences between groups. All values are shown as mean ± SE and a *p* value of < 0.05 was considered to indicate a significant difference.

## Results and discussion

### Synthesis and characterization of amphiphilic ABA type triblock copolymers

**Figure 1:**
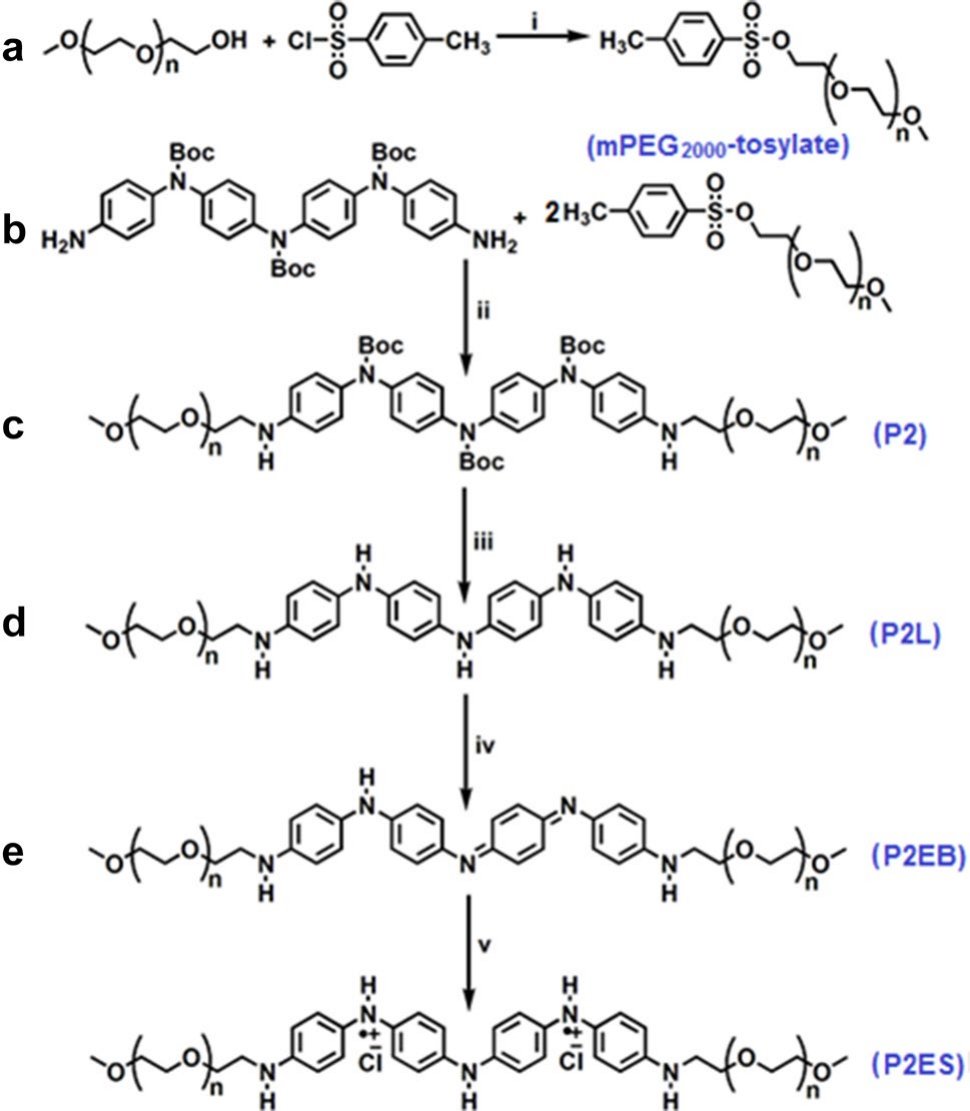
Synthesis of amphiphilic ABA type triblock copolymers containing secondary amine (NH) linkage (**a–e**). (i) NaOH, THF, 0–5 °C, 4 h; (ii) DMAP, ET_3_N, DMSO, 65 °C, 24 h; (iii) TFA, DCM, 0 °C to room temperature; (iv) APS, 2 M HCl, 2 M NH_4_OH, room temperature, 4 h; (v) 1 M HCl, room temperature, 24 h. P2 (n = 44).

A TANI-based ABA type coil-rod-coil triblock copolymer containing amine linkage P2 was successfully synthesized. The triblock copolymers were synthesized in two steps as shown in Fig. 1. In the first step the hydroxyl end group of mPEG_2k_ was modified to mPEG_2k_-tosylate^28^, and in the second step, the coupling of TANI with modified mPEG_2k_^17^ was performed. After the synthesis of the boc-protected triblock copolymers, deprotection was carried out using TFA to assess the electroactive nature of the triblock copolymers induced by the TANI segment^17^. Deprotection resulted in the fully reduced leucoemeraldine (LEB) state of TANI, which was half oxidized to its most stable emeraldine (EB) state^17^. Doping of the EB state of the copolymers resulted in conducting emeraldine salt (ES) state^17^. The resulting synthesized triblock copolymers were predried under vacuum overnight and structurally characterized by utilizing FTIR, ^1^H NMR, ^13^C NMR, and mass spectrometric studies.

**Figure 2:**
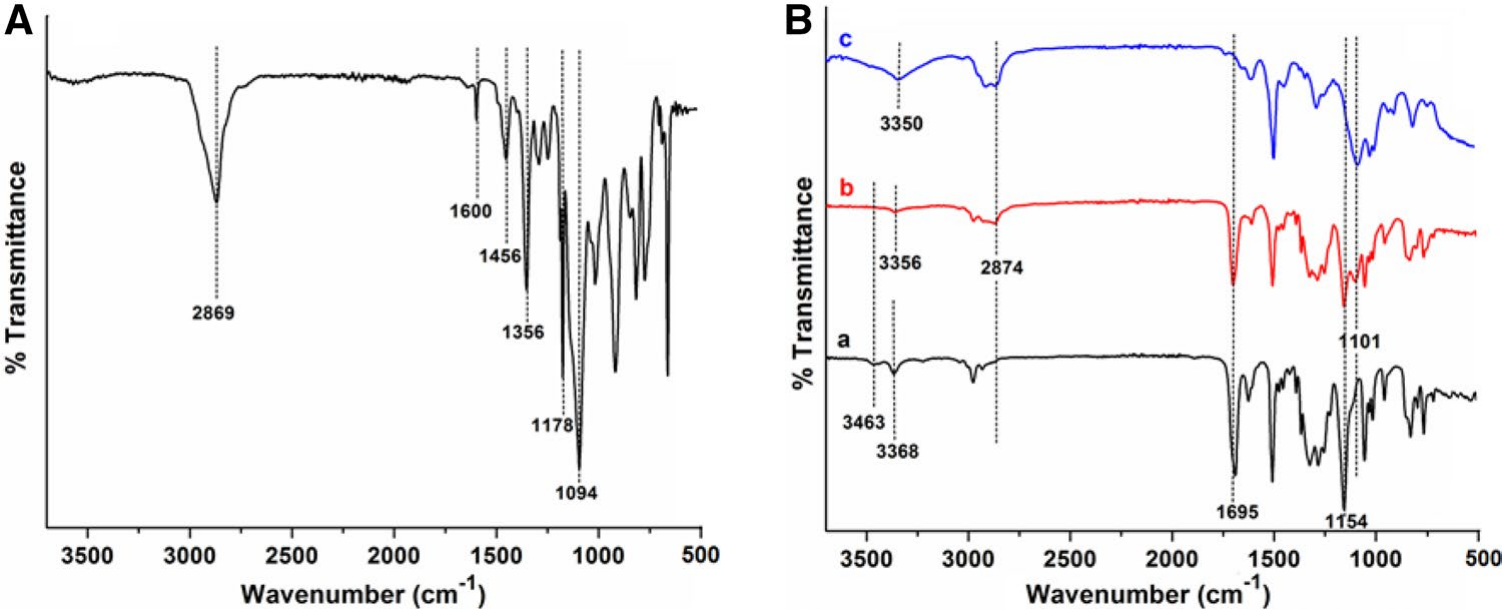
FTIR spectra (**A**) mPEG_2k_-tosylate, and (**B**) TANI (a), P2 (b), and P2L (c).

The FTIR spectra for the synthesized triblock copolymer are shown in Fig. 2. The FTIR spectrum for mPEG-tosylate is shown in Fig. 2A. Characteristic C–H and C–O–C stretching vibrations for monomethoxy capped-PEG moiety appeared at 2869 cm^−1^ and 1094 cm^−1^, respectively. The O=S=O asymmetric and symmetric stretch occurred at 1356 cm^−1^ and 1178 cm^−1^, respectively. The C=C stretching bands for the aromatic ring appeared at 1600 cm^−1^ and 1456 cm^−1^^34^. Figure 2B shows the FTIR spectrum for P2. We noted appearance of the stretching vibration band at 3356 cm^−1^ for the secondary amine N–H in the spectrum of P2 (Fig. 2B(b)). In contrast, the disappearance of stretching bands for the primary NH_2_ moiety at 3463 cm^−1^ and 3368 cm^−1^ as shown in Fig. 2B(a) confirmed the formation of the product. The presence of mPEG_2k_ in the product was assessed by the stretching vibrations at 2874 cm^−1^ and 1101 cm^−1^ for C-H and C–O–C moieties respectively. Whereas, deprotection of P2 resulted in the disappearance of characteristic peaks for the boc group at 1695 cm^−1^ and 1154 cm^−1^ for ester carbonyl and C–O–C moieties (Fig. 2B(c)). We noted a broad stretching vibration for NH moieties of the TANI segment in P2L at 3350 cm^−1^.

**Figure 3:**
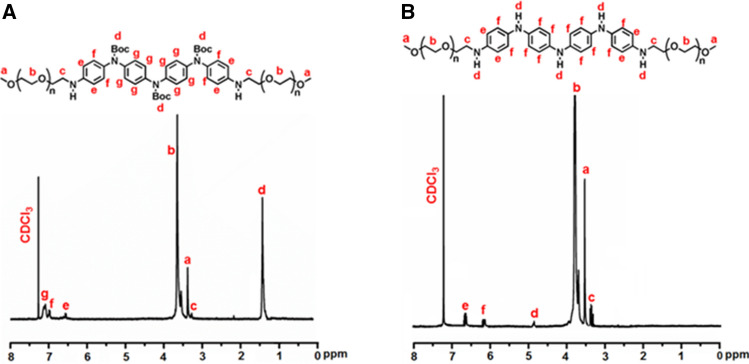
^1^H NMR spectra (**A**) P2, and (**B**) P2L.

The synthesized amphiphilic triblock copolymers were further structurally characterized by ^1^H NMR and ^13^C NMR techniques. The ^1^H NMR spectra of the synthesized amphiphilic ABA-type triblock copolymers containing secondary amine linkage are shown in Fig. 3. In all the ^1^H NMR spectra, the CDCl_3_ solvent signal appears at 7.28 ppm. The ^1^H NMR of mPEG_2k_-tosylate is shown in Fig. S1. The methylene protons of mPEG_2k_ (^e^CH_2_) signals appeared at 3.61 ppm while those attached with the tosyl group are deshielded to 4.14 ppm due to attachment with an electronegative oxygen atom. The end-capped methoxy protons signal can be observed at 3.8 ppm. The aromatic protons attached at ortho position to tosyl (^c^CH) and methyl (^b^CH) moieties showed signals at 7.78 ppm and 7.32 ppm, respectively. The methyl protons (^a^CH_3_) showed resonance at 2.43 ppm^35^. The ^1^H NMR spectrum of P2 is displayed in Fig. 3A. The boc-protected TANI segment aromatic protons signals (^e,f,g^CH) in P2 appeared at 7.09–6.53 ppm. The signals for the mPEG_2k_ segment can be observed at 3.65 ppm and 3.38 ppm for methylene protons (^b^CH_2_) and terminal methoxy protons (^a^OCH_3_), respectively. The presence of secondary amine linkage was assessed by observing the disappearance of the peak at ~ 5.0 ppm for the NH_2_ protons of TANI. The deprotection of P2 resulted in a fully reduced TANI segment in P2L as shown in Fig. 3B. The aromatic protons displayed upfield resonance at 6.78 ppm while signals at 1.42 ppm disappeared which confirmed the removal of boc moiety.

The ^13^C NMR spectra of the synthesized triblock copolymers are presented in Fig. S2. A resonance signal at 77 ppm was observed in all the ^13^C NMR spectra, and was assigned to CDCl_3_. In all the ^13^C NMR spectra the resonance signals for methylene and end methoxy carbons were observed at 65–72 ppm and 59 ppm, respectively. The aromatic carbons of the TANI segment were observed in the region of 114–131 ppm. In boc-protected triblock copolymers, the ester carbonyl carbon of the boc moiety appeared at 155 ppm in P2 while this signal disappeared upon deprotection of P2 to P2L.

GPC measurements also showed the successful synthesis of P2L (Fig. S3). The absolute molecular weight of TANI-LEB segment is 381 with MALDI-TOF detection, as reported earlier^17^. mPEG_2k_ is a commercially available polymer with a known molecular weight of 2000 g/mol. The molecular weight of P2L calculated to be 4381 by using the formula “mPEG_2k_-TANI-mPEG_2k_, fell very close to GPC value of 4172, with a polydispersity index (PDI) of 1.26 (Fig. S3).

### Electrochemistry of amphiphilic ABA type triblock copolymers

**Figure 4:**
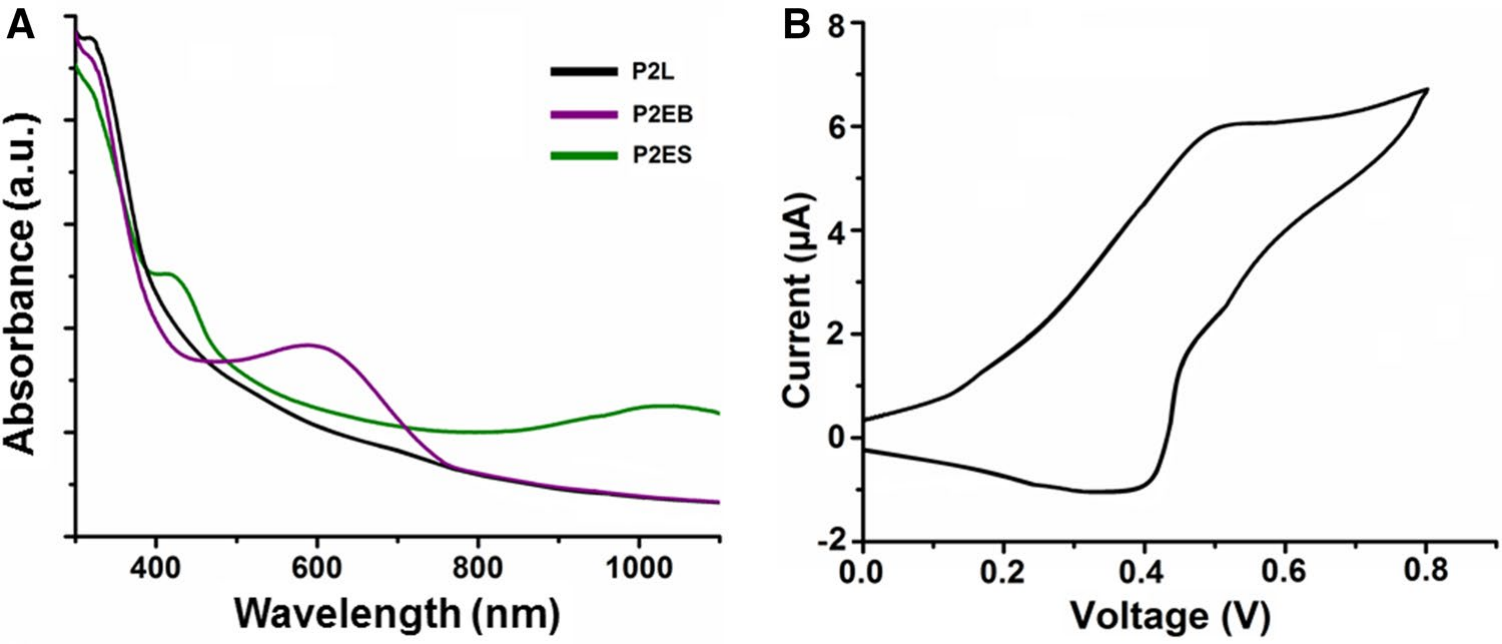
UV–Vis–NIR spectra of amphiphilic ABA type triblock copolymers (**A**), and cyclic voltammogram of P2L (**B**).

The synthesized amphiphilic ABA-type triblock copolymers showed an electroactive nature due to the inclusion of the redox-active TANI segment. We monitored the electroactivity of P2L using cyclic voltammetry and UV–Vis–NIR spectroscopy techniques. Figure 4A shows the UV–Vis–NIR spectra of P2 in different oxidation states. Identical spectra were observed for its low molecular weight analog^17^. The leucoemeraldine state (P2L) showed a single peak at 321 nm for the π–π* transition of benzenoid rings (Fig. 4A, curve 1). The oxidation of the leucoemeraldine state (P2L) to emeraldine state (P2EB) showed a hypsochromic shift in the peak at 321–309 nm, along with the appearance of a peak at 587 nm that can be assigned to the n–π* transition of the benzenoid ring to quinoid form (Fig. 4A, curve 2). P2EB was doped with 1 M HCl, which resulted in the typical appearance of peaks at 420 nm and 980 nm (Fig. 4A, curve 3) along with the disappearance of the peak at 587 nm. These peaks were assigned to the polaron–π* transition and π–polaron absorption, respectively. Figure 4B displayed the cyclic voltammogram of P2L. The characteristic single reversible redox peak was observed at a mean peak potential (E_1/2_) of 0.45 V depicting the conversion of the leucoemeraldine base (LEB) to the emeraldine base (EB) state^36^.

### Air oxidation behavior of P2L

**Figure 5:**
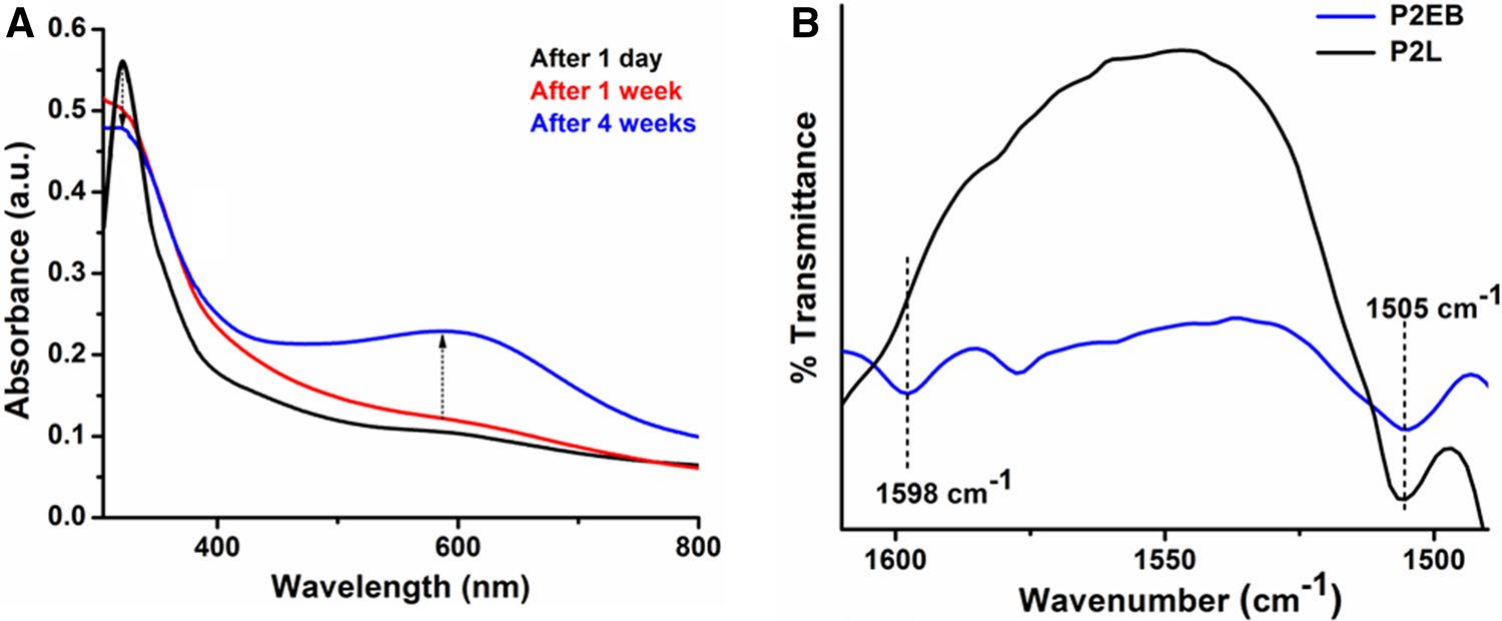
Air oxidation of P2L. UV–Vis−NIR spectra (**A**), and FTIR spectra (**B**).

The LEB state of TANI can undergo oxidation in air while at room temperature^11^. The air oxidation behavior of the amphiphilic triblock copolymer in the LEB state (P2L) was monitored using UV–Vis–NIR and FTIR spectroscopic techniques. The aqueous solution of P2L was kept in the air and checked for its air oxidation over 4 weeks. Figure 5A shows the UV–Vis–NIR spectra of the air oxidized sample of P2L. These spectra depicted the occurrence of air oxidation of the LEB state of P2L with the appearance of characteristic oxidation peak for the LEB to EB state at 580 nm.

The air oxidation behavior was further confirmed by running their FTIR spectra before and after air oxidation (Fig. 5B). In the LEB state of the triblock copolymer, all the aromatic rings are in a benzenoid form showing a strong aromatic stretching vibration band at 1505 cm^−1^ and a weak stretching vibration band at 1605 cm^−1^. In contrast, the FTIR spectrum of the same sample recorded after 4 weeks showed two aromatic stretching vibration bands at 1505 cm^−1^ and 1605 cm^−1^ of equal intensity that represented the partial conversion of benzenoid rings to quinoid form36.

Hence FTIR spectroscopic studies also revealed the air oxidation behavior of P2L.

### Acid/base behavior of P2EB

The EB state of the amphiphilic ABA type triblock copolymer P2EB was also investigated for its acid–base sensitivity using UV–Vis–NIR spectra of as shown in Fig. S4. The aqueous solution of P2EB was doped with 0.1 M HCl. An immediate change in color (from purple to green) was observed depicting the conversion of the EB state to ES state of the TANI segment in the synthesized amphiphilic triblock copolymer. Figure S4 (curve 1) shows the appearance of peaks in the ranges of 420 nm and 950–980 nm that can be ascribed to the polaron-π^*^ transition and π–polaron transitions, respectively^37^. The green-colored acid-doped solution (P2ES) was dedoped by adding 0.1 M NH_4_OH. Again, an instant change in color from green to purple was observed. The UV–Vis–NIR spectra (Figure S4 curve 2) displayed the disappearance of peaks at 420 nm and 980 along with the presence of a characteristic peak at ~ 600 nm showing the conversion of ES state to the EB state of TANI. The conversion of the redox states in acidic and basic media showed good acid–base sensitivity of the synthesized triblock copolymer.

### Aqueous self-assembly

The synthesized amphiphilic ABA type triblock copolymer (PEG_2k_–TANI–PEG_2k_) has two segments i.e., hydrophobic and rigid TANI segments and hydrophilic and flexible mPEG segments. Based on the amphiphilic nature of the triblock copolymers, these can be self-assembled in water forming the mPEG corona surrounding the TANI core^38^. The aqueous self-assembly behavior of the amphiphilic ABA type triblock copolymer was investigated using various techniques. The change in size and morphology of the self-assembled structures of the triblock copolymers with and without the external stimuli were also taken into consideration. The initial aggregate formation was anticipated by recording the ^1^H NMR spectrum in D_2_O. Figure S5 shows the ^1^H NMR spectra performed in D_2_Oand CDCl_3_. All the respective signals of the triblock copolymer were seen in the ^1^H NMR spectrum measured in CDCl_3_ (Fig. S5A) showing its complete dissolution in CDCl_3_. The ^1^H NMR spectrum recorded in D_2_O (Fig. S5B), exhibited only signals for the mPEG segment. The TANI signals were obscured indicating that the mPEG corona surrounded the TANI core^11,39^.

### Critical micelle concentration (CMC)

The critical micelle concentration (CMC) was measured using fluorescence probe experiments. In this method, a hydrophobic fluorescent dye was employed that is susceptible to changes in the solvent properties noticeable in its fluorescence spectrum^39^. Pyrene a fluorescence probe was used and from the emission spectrum, the intensity ratios (*I*_1_:*I*_3_) of the peaks at 376 nm and 392 nm, respectively, were plotted vs. the logarithm of the concentration of species in the solution^40^. The CMC value can be determined from the intersection of the best-fit lines. This intersection point shows the minimum copolymer concentration required for the formation of stable micelles. When this concentration is attained, the pyrene, a hydrophobic molecule, is incorporated into the hydrophobic core of the aggregates^41^. The CMC value of P2L was determined by fluorescence probe experiments as shown in Fig. S6. P2L showed a CMC value of 0.25 mM greater than the previously found CMC value (0.02 mM) of its lower molecular weight analog (PEG_350_–TANI–PEG_350_)^17^. This may be attributed to the effect of the molecular weight of mPEG. In the low molecular weight analog of P2L (PEG_350_-TANI-PEG_350_), the hydrophobic character of the TANI segment is increased due to the lower molecular weight of the mPEG hydrophilic segment. This increased hydrophobic character leads to more aggregation of TANI segments via hydrogen bonding and π–π interactions, thus lowering the CMC according to the reported literature^11^.

### Tuning of CMC of P2L without external stimuli

**Figure 6:**
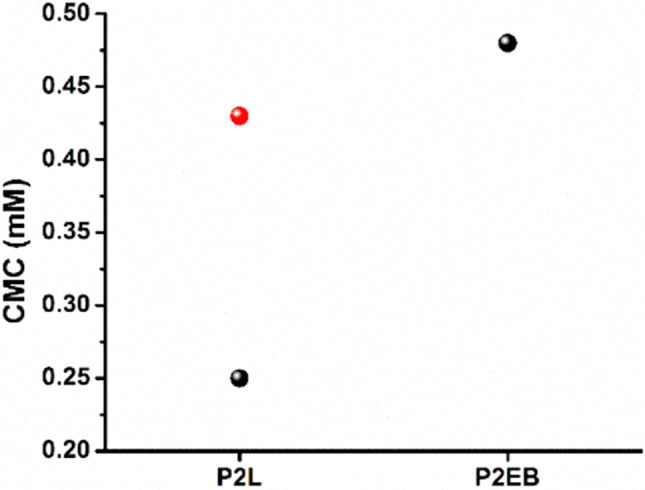
CMC data of P2L and P2EB after 1 day (black balls), and after 4 weeks (red balls).

The oxidation state of the TANI segment in amphiphilic triblock copolymers can be utilized to tune their CMC without any external stimuli^11^. Fluorescence spectroscopy was used to investigate the CMC of P2L in LEB and EB states. Anticipating aggregate formation and stability without any external stimulus, triblock copolymer in its LEB state was kept in the air at room temperature for 4 weeks. The oxidation of P2L was monitored by UV–Vis–NIR spectroscopic studies (Fig. 5) as discussed above. The CMC of the oxidized samples was measured after 4 weeks (Fig. 6). The LEB state has lower CMC than the EB state of the triblock copolymer. This is most likely due to the increased hydrogen bonding on affording five NH groups as hydrogen bond donors by the LEB state while the EB state has only three^36^. The CMC value of P2L obtained after 4 weeks was increased and found very close to P2EB demonstrating that oxidation of the LEB state had occurred. This study showed the tunable CMC values of P2L copolymer based on the oxidation phenomenon, which can be promising in drug delivery applications.

### Size and morphology of triblock copolymer

**Figure 7:**
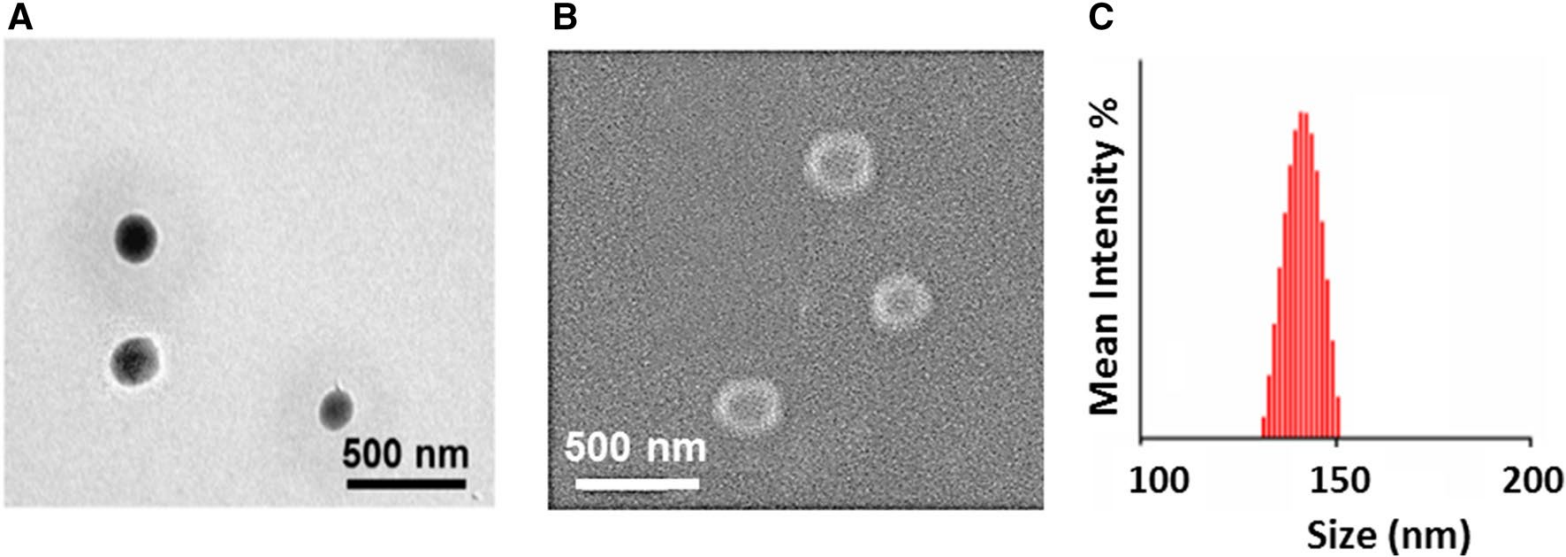
Aqueous self-assembly of the amphiphilic triblock copolymer P2L. TEM image (**A**); SEM image (**B**) and, particle size distribution by DLS (**C**).

For aqueous self-assembly of nonionic amphiphilic triblock copolymer, a sample solution was prepared in double-distilled water^42^. To study the effect of oxidation state and doping on the size and morphology, the samples were prepared in their LEB, EB, and ES states. Figure 7 shows the TEM, and SEM images of the P2L aggregates formed in an aqueous solution. P2L showed the spherical aggregates with an average size of 140 nm (Fig. 7A,B). An increase in the size of the spherical aggregates was observed on increasing the molecular weight of mPEG (from 350 to 2000 g/mol) compared to the previously reported analog of P2L^17^. This may be attributed to the tuned hydrophobic to hydrophilic ratio. The low molecular weight mPEG (mPEG_350_) segment, compared to high molecular weight (mPEG_2k_) segment, imparts less hydrophilic character in the amphiphilic triblock copolymers; hence, the hydrophobicity of the TANI segment increases. This hydrophobicity causes the exclusion of some water molecules in the vicinity of the rigid aromatic TANI regions. Therefore, more TANI molecules come closer to each other developing strong hydrogen bonding between the amine moieties and the increased π–π interactions of aromatic rings resulting in shrinkage of the size of the aggregates^11^. The DLS study also showed the presence of aggregates of size 140 ± 10 nm (Fig. 7C), complimenting the results obtained from the SEM analysis. According to the literature, this small change in size is due to the drying effect of the solvent^43^.

### Effect of changing oxidation state on the aggregation of amphiphilic ABA type triblock copolymer

**Figure 8:**
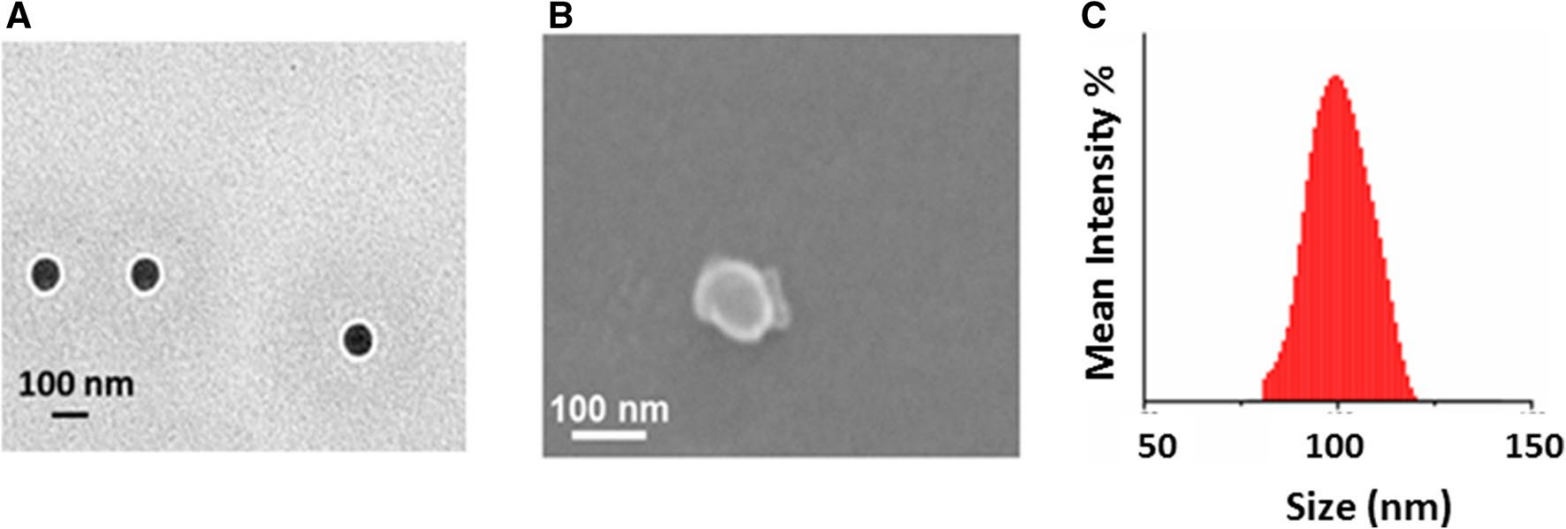
Aqueous self-assembly of P2EB. TEM image (**A**), SEM image (**B**) and particle size distribution by DLS (**C**).

The change in the oxidation state of the TANI segment can also affect the aggregation behavior of the amphiphilic triblock copolymer. For example, P2L oxidized to its EB state (P2EB showed the same spherical morphology with a decrease in the size of the aggregates up to 90 nm (Fig. 8A). This decrease in size can be attributed to the additional structure directing amine–imine hydrogen bonding as reported earlier^44^. DLS studies also showed similar hydrodynamic diameters (90 ± 10 nm) objects in aqueous solution (Fig. 8C), which agrees very well with the SEM measurements (Fig. 8B).

### Effect of pH change on the aggregation of amphiphilic ABA type triblock copolymer

**Figure 9:**
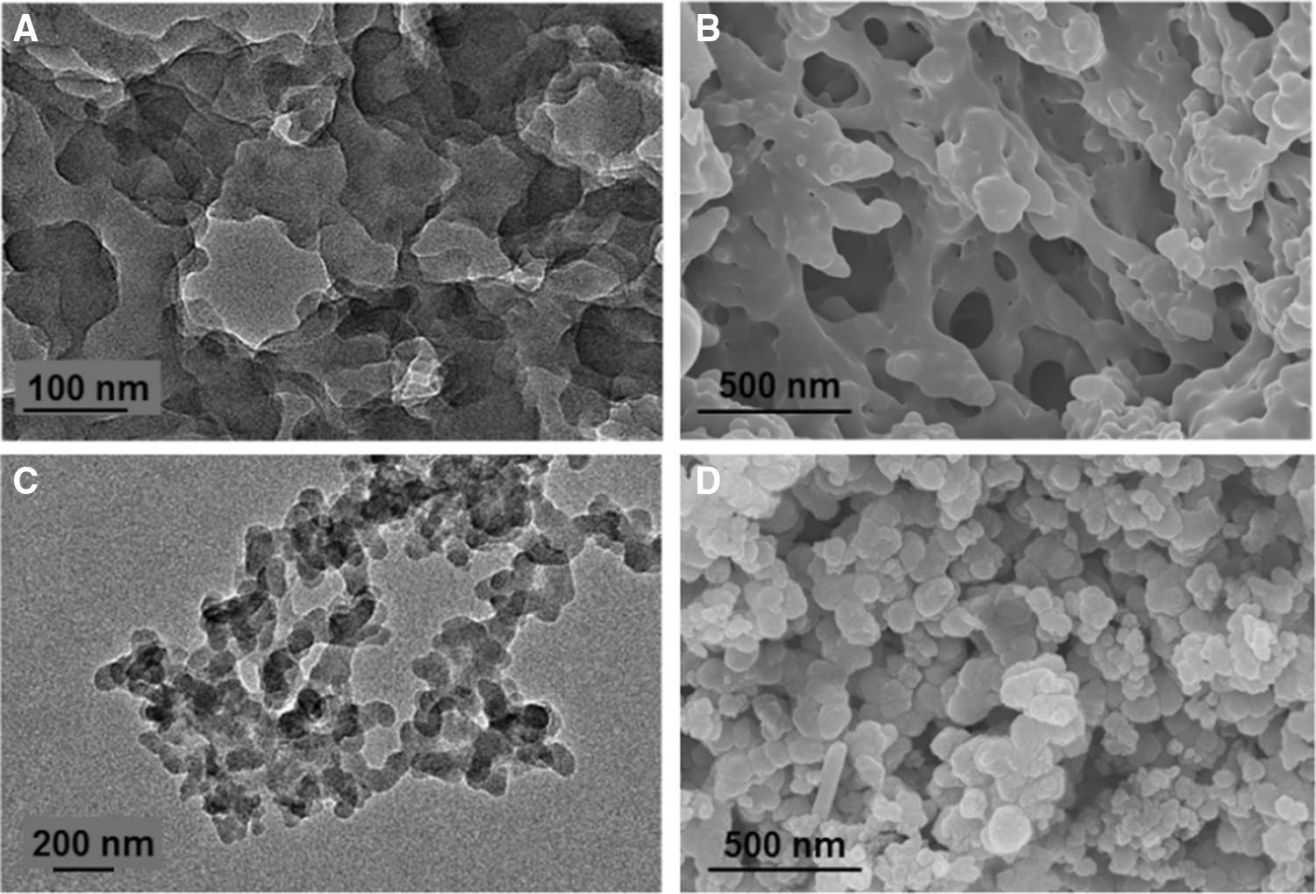
Tunable self assembly of P2EB. In acidic medium (0.1 M HCl, pH 1.1), TEM image (**A**), and SEM image (**B**); In basic medium (0.1 M NH_4_OH, pH 11.1), TEM image (**C**), and SEM image (**D**).

Our data showed that self-assembled aggregates were sensitive to acid/base medium. Thus, we investigated the aqueous self-assembly of P2L in media with different pH values using TEM, SEM, and DLS methods. The sample solution was prepared by directly dissolving the P2EB in a 1 M HCl (pH 1.1) solution. This acidic doping of the P2EB to P2ES and dedoping by using 1 M NH_4_OH (pH 11.1) was confirmed by UV–Vis–NIR spectra as shown in Fig. S4. The previously reported low molecular weight analog of P2L showed self-assembled colonies of spherical aggregates of 103 nm size in an acidic medium studied by TEM, SEM, and DLS analyses^17^. The amphiphilic triblock copolymer (P2EB) having a high molecular weight of mPEG (M_w_ = 2000 g mol^−1^) showed no spherical aggregate formation in acidic medium (pH 1.1), but rather a soluble network was observed as investigated by the TEM and SEM analyses (Fig. 9A,B).

Furthermore, the DLS studies confirmed the absence of aggregates, which could be due to increased hydrophilicity on acidic doping showing the same behavior as reported earlier^45^. This could be advantageous because it allows the TANI segment to express full antioxidant potential compared to the reduced segment due to the internal core. The dedoping of P2L using 1 M NH_4_OH again showed aggregates of comparable size with P2EB (Fig. 9C,D). This switching in self-assembled acid/base media structures may be a promising approach in potential drug delivery applications.

### Cytotoxicity analysis in human whole blood samples (hemolytic assay)

**Figure 10:**
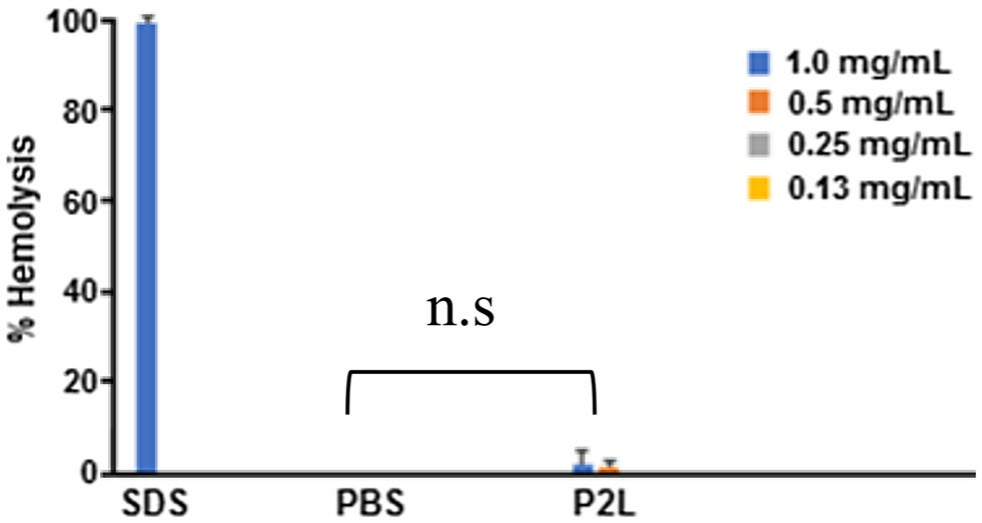
In vitro cytotoxicity analysis (Hemolytic assay) of P2L performed in RBCs obtained from healthy individuals showed no toxicity, compared to PBS (n = 3).

If drug carriers are toxic, this can trigger undesired cellular injuries and hemolysis in treated subjects. To investigate whether P2L is nontoxic, the hemolytic activity of the amphiphilic triblock copolymer P2L was measured. The hemolytic activity of P2L was measured using four different concentrations i.e. 1 mg/mL, 0.5 mg/mL, 0.25 mg/mL, and 0.13 mg/mL. Sodium dodecyl sulfate (SDS), an emulsifying agent and known to cause hemolysis at very low concentrations, was used as a positive control. We found that P2L caused only 1% hemolysis at a 1 mg/mL concentration compared to 98% hemolysis by SDS at the same concentration (Fig. 10). Wherease, P2L 0.25 mg/mL and 0.13 mg/mL showed no toxicity when compared to PBS. These data suggest that the P2L is nontoxic when interacting with whole blood.

### Computational analysis for binding of triblock copolymer and dexamethasone

**Figure 11:**
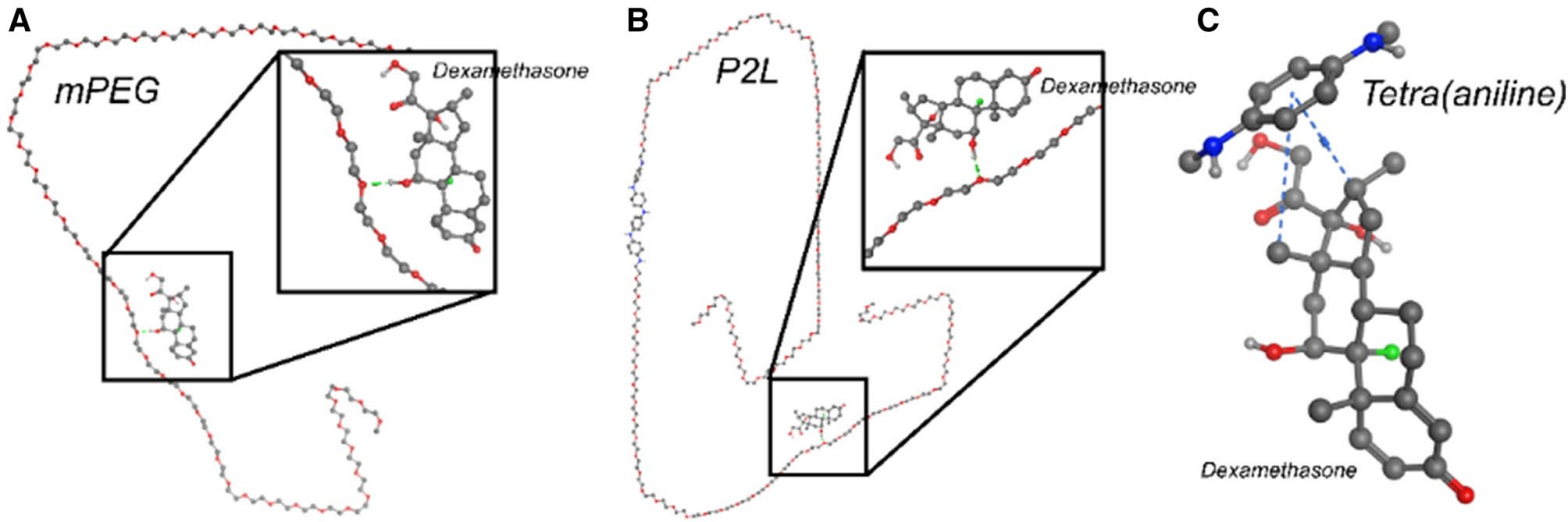
Interaction of P2L with the dexamethasone. The docked conformation of (**A**) dexamethasone and mPEG_2k_ (**B**) dexamethasone and P2L, and (**C**) dexamethasone and TANI. The ball and stick model represent both dexamethasone and P2L. Blue dash and green lines indicate the hydrogen and phi-stacking interaction.

We next performed molecular docking to understand the binding interaction between P2L and its constituents with dexamethasone. Consequently, it was observed that dexamethasone binds with P2L as shown in Fig. 11. In the case of both mPEG_2k_ and P2L, we have noted single hydrogen bond interaction, while in the case of TANI, it was pi-stacking interaction. Our docking calculation further predicts binding energies of − 5.4356 kJ/mol for mPEG_2k_ − 4.9232 kJ/mol for P2L, and − 5.5354 kJ/mol for TANI.

These results suggest that P2L could be used as a drug carrier system for glucocorticoids such as dexamethasone. Dexamethasone is a small and hydrophobic molecule that binds to the GC receptors and is widely used to treat cancers and inflammation, despite its severe side effects on bone and other tissues^47^. Systemic administration of dexamethasone is also known to cause insulin resistance, therefore, delivery of dexamethasone encapsulated in P2L would be of great clinical interest under different pathological conditions.

### P2L, a potential drug delivery system for clinically used drug dexamethasone

As a proof of concept, we also investigated the potential of P2L as a drug delivery system for dexamethasone with and without electrical stimulation. We observed that dexamethasone showed absorbance at 242 nm (Fig. S7a) that was masked with the addition of P2L after 15 min (Fig. S7b) and finally disappeared after 30 min (Fig. S7c), indicating that the dexamethasone was indeed encapsulated within the P2L aggregate core.

**Figure 12:**
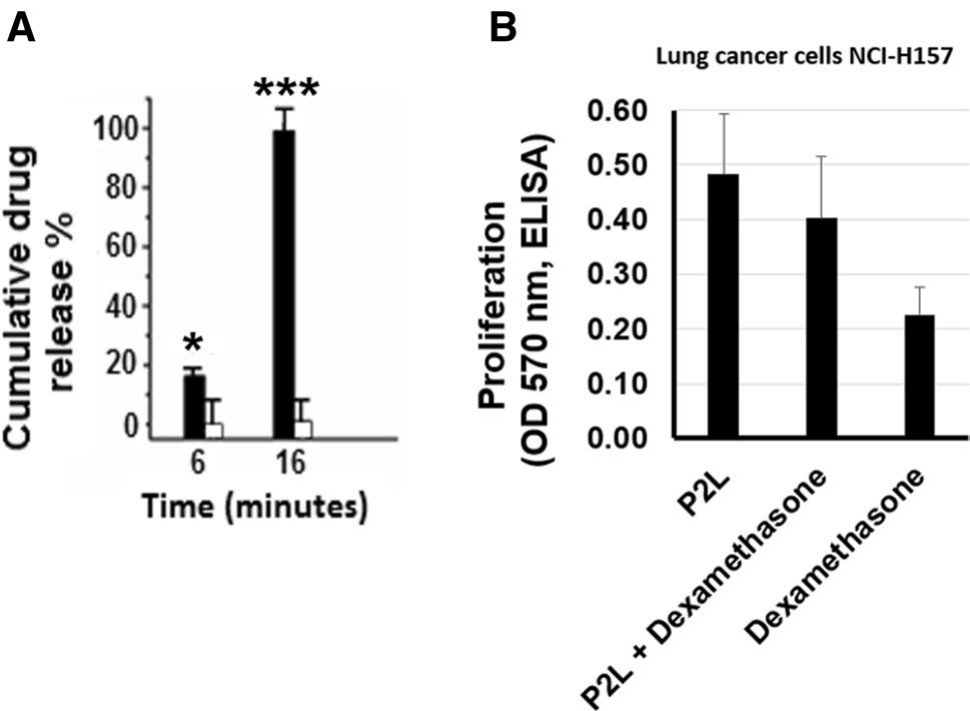
Dexamethasone release after 6 and 16 min in the presence (black bars) and absence (white bars) of electrical stimulation using UV–Vis spectroscopy (n = 3) (**A**). Lung cancer cells NCI-H157 were exposed to P2L, dexamethasone encapsulated in P2L (P2L+ Dexamethasone) and dexamethasone (3 µM) for 48 h, a trend towards decreased cells proliferation in P2L + Dexamethasone (17% vs P2L, non-significant, n = 3) was observed. Dexamethasone alone significantly suppressed proliferation (p < 0.01), compared to P2L alone (**B**). *p < 0.05, ***p < 0.001.

We next measured the release of dexamethasone by using UV–Vis spectroscopy at 242 nm after applying the oxidation potential (+ 0.5 V) to P2L. Figure 12A shows 16% release of the dexamethasone after 6 min, followed by 99% release after 16 min. The observed release (Fig. 12A) is due to the oxidation of the TANI segment in P2L achieved through the conversion of the LEB state to the EB state, shrinking the size of the aggregates as discussed in “Effect of changing oxidation state on the aggregation of amphiphilic ABA type triblock copolymer”. This decrease in the size of the aggregates accompanies the release of the encapsulated drug. We also monitored the release of dexamethasone in the absence of electrical stimulation (passive drug release), only 5% dexamethasone was released after 6 and 16 min, complimenting with the air oxidation behavior of P2L as described in “Air oxidation behavior of P2L”.

Normal cells have an extracellular pH value of approximately 7.4, whereas the pH of cancer cells varies between 6.7 and 7.1^46^. In our experimental model NCI-H157 human lung cancer cells, the extracellular pH value was 6.8 which provided us with the opportunity to investigate whether low pH can trigger an encapsulated drug as described in “Effect of pH change on the aggregation of amphiphilic ABA type triblock copolymer”. Human lung cancer cells with extracellular pH value of 6.8 were exposed to P2L+ dexamethasone for 48 h. Therefore, assuming drug release in response to extracellular low pH value, we expected decreased cell proliferation, as dexamethasone is known for its antiproliferative effects. Interestingly, lung cancer cells co-incubated with P2L+ dexamethasone showed a trend toward reduced cell proliferation by 17%, although this effect was non-significant compared to P2L alone (Fig. 12B). These data compliment the effect of pH change on the behavior of P2L as described in “Effect of pH change on the aggregation of amphiphilic ABA type triblock copolymer”.

## Conclusions

**Figure 13:**
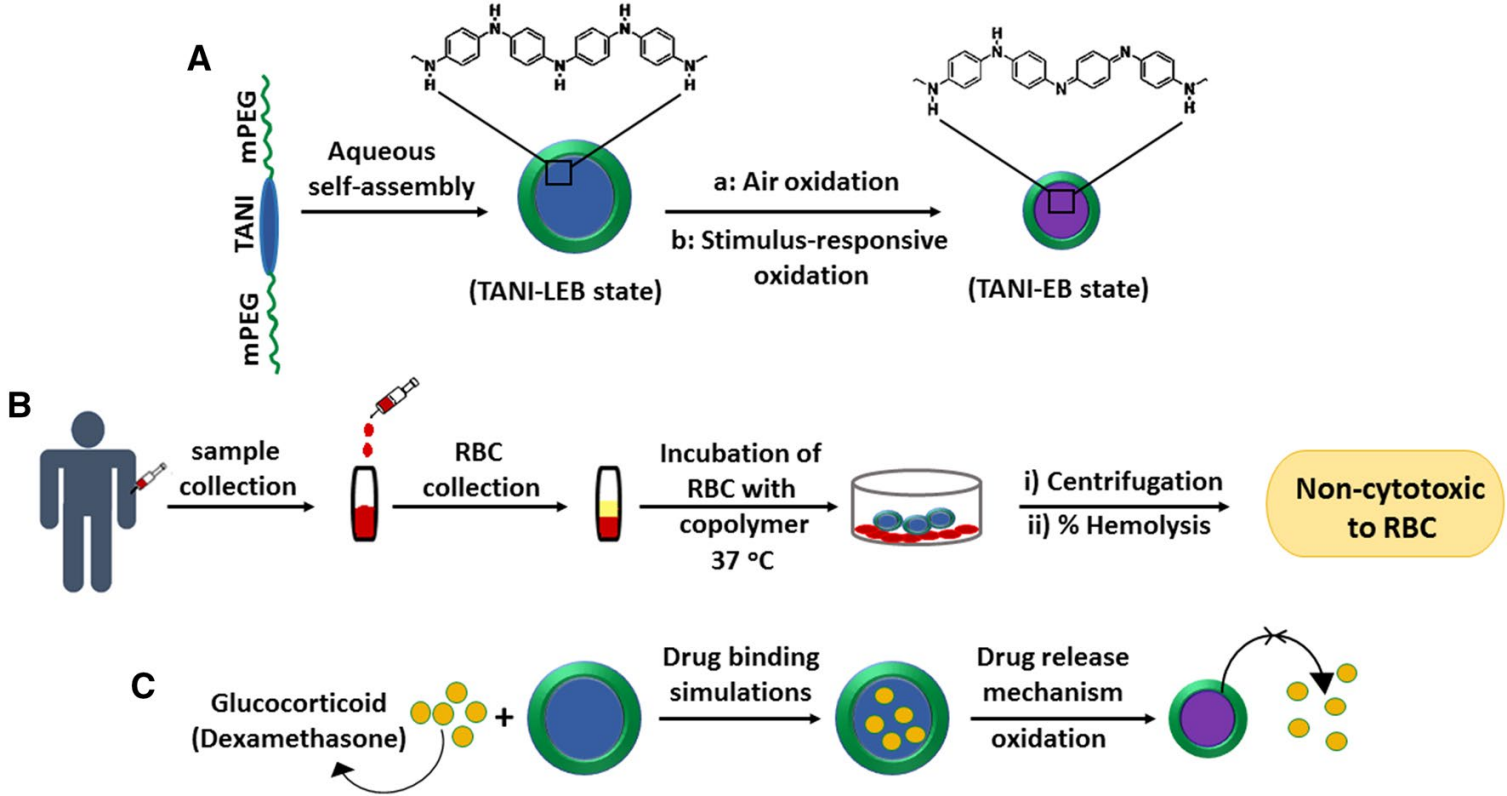
Synthesis, testing cytotoxicity, drug encapsulation (uptake/release), and computational analysis of triblock copolymer P2L. (**A**) Tunable aqueous self-assembly of the synthesized ABA-type triblock copolymer P2L. (**B**) Cytotoxicity analysis of copolymer P2L in the human blood samples under in vitro conditions. (**C**) Experimental and computational studies show the binding and potential delivery/release of dexamethasone by using copolymer P2L.

In summary (Fig. 13), we demonstrated the synthesis of a new class of amphiphilic triblock copolymer P2L containing mPEG_2k_ that exhibits tunable self-assembly and nontoxic behavior when tested in human blood samples in vitro. We also showed that P2L encapsulates clinically used drug dexamethasone, which can then be released by electrical stimuli and low pH, thereby opening potential use in different pathological conditions such as cancer and inflammation. These findings further advance the knowledge of developing nontoxic, safe supramolecules as DDSs and their use in implantable drug delivery systems/devices with precise drug administration and controlled release.

## Supplementary Information

Supplementary Information.
