# Supplementary material for: A unique amphiphilic triblock copolymer, nontoxic to human blood and potential supramolecular drug delivery system for dexamethasone
Source: Sci Rep. 2021 Nov 2;11:21507. doi: 10.1038/s41598-021-00871-w (PMC8563740; doi:10.1038/s41598-021-00871-w)
Supplement: Supplementary file 1 — Supplementary Information. [file 41598_2021_871_MOESM1_ESM.pdf]

Supplementary Information

A unique amphiphilic triblock copolymer, nontoxic to human blood and potential supramolecular drug delivery system for dexamethasone

Irrum Mushtaq[a], Zareen Akhter[a], Muhammad Farooq[a], Farukh Jabeen[b], Ashfaq Ur Rehman[c], Sadia Rehman[d], Sidra Ayub[e], Bushra Mirza[e], Muhammad Siddiq[a], Farasat Zaman[f] *

[a]Department of Chemistry, Quaid-i-Azam University Islamabad 45320, Pakistan

[b]Department of Chemistry and Biochemistry, Laurentian University, 935 Ramsey Lake Road, Sudbury, ON, Canada P3E 2C6

[c]Department of Pathophysiology, Key Laboratory of Cell Differentiation and Apoptosis of Chinese Ministry of Education, Shanghai Jiao Tong University, School of Medicine, Shanghai 200025, China.

[d]Institute of Biomedical and Genetic Engineering, Islamabad, Pakistan

[e]Department of Biochemistry, Quaid-i-Azam University Islamabad 45320, Pakistan

[f]Department of Women's and Children's Health, Karolinska Institutet and Pediatric Endocrinology Unit, Karolinska University Hospital, SE-171 74 Solna, Sweden

*Corresponding author: farasat.zaman@ki.se , Karolinska University Hospital, SE-171 74 Solna, Sweden

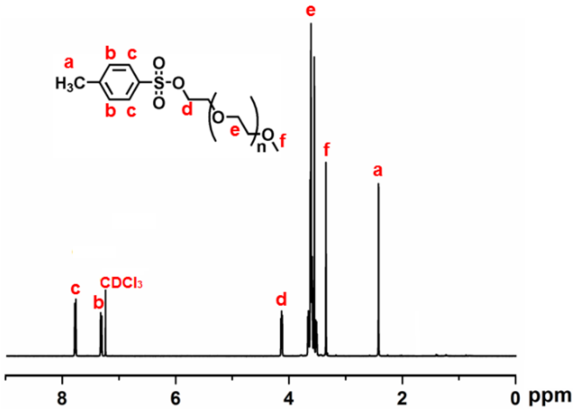

Figure S1. ^{1}H NMR spectrum of PEG_{2k}-tosylate

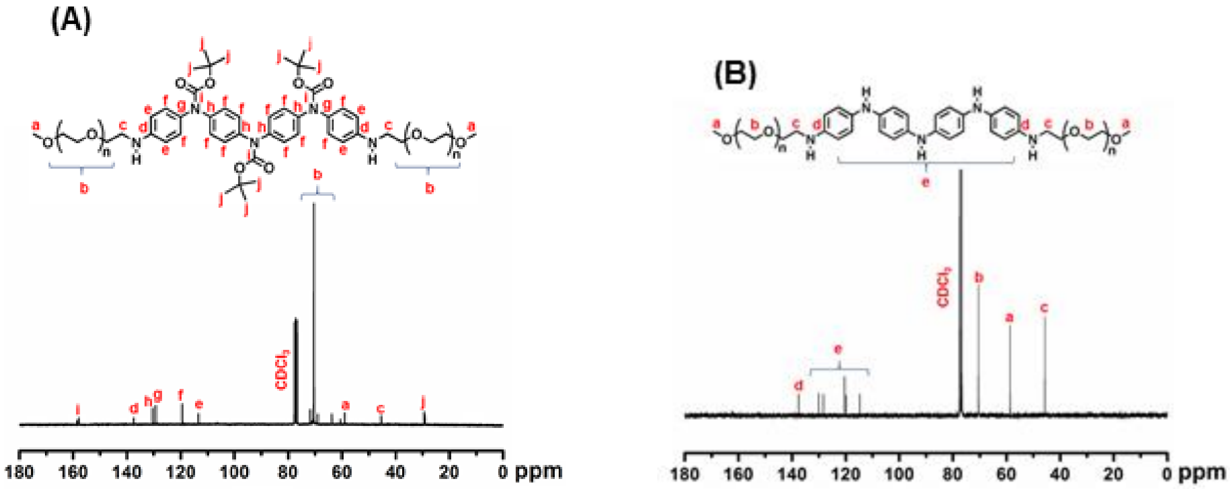

Figure S2. ^{13}C NMR spectra of P2 (A), and P2L (B).

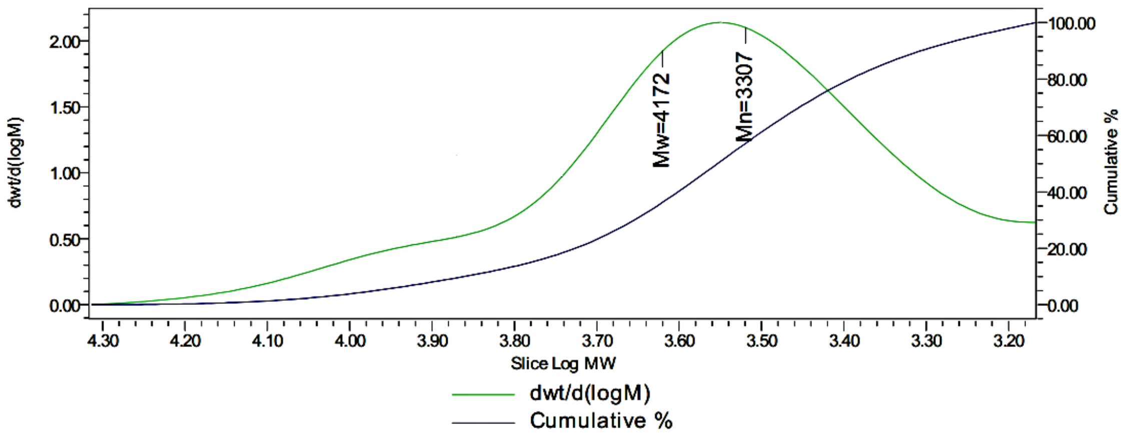

Figure S3. Gel permeation chromatogram showing M_w and M_n of P2L.

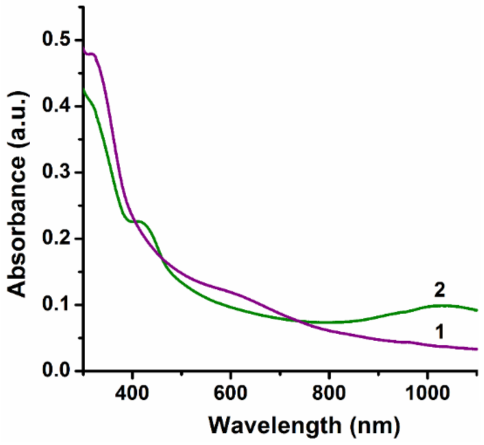

Figure S4. UV-Vis-NIR spectra of P2EB (1) in acidic medium (1M HCl), and (2) in basic medium (1M NH_4OH)

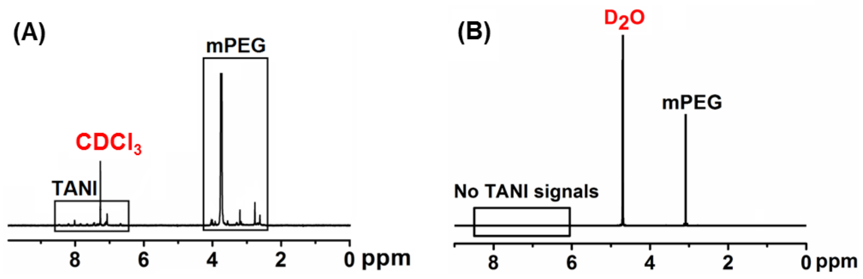

Figure S5. Aqueous self-assembly examination by ^{1}H NMR spectra of P3L in (A) $CDCl_3$ and, (B) D_2O.

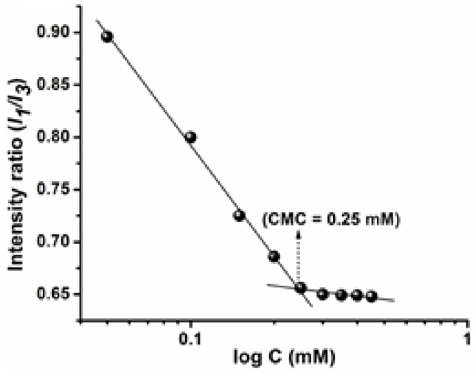

Figure S6. Fluorescence probe experiment. A plot of pyrene I_1:I_3 ratio vs. logarithmic concentration of P2L.

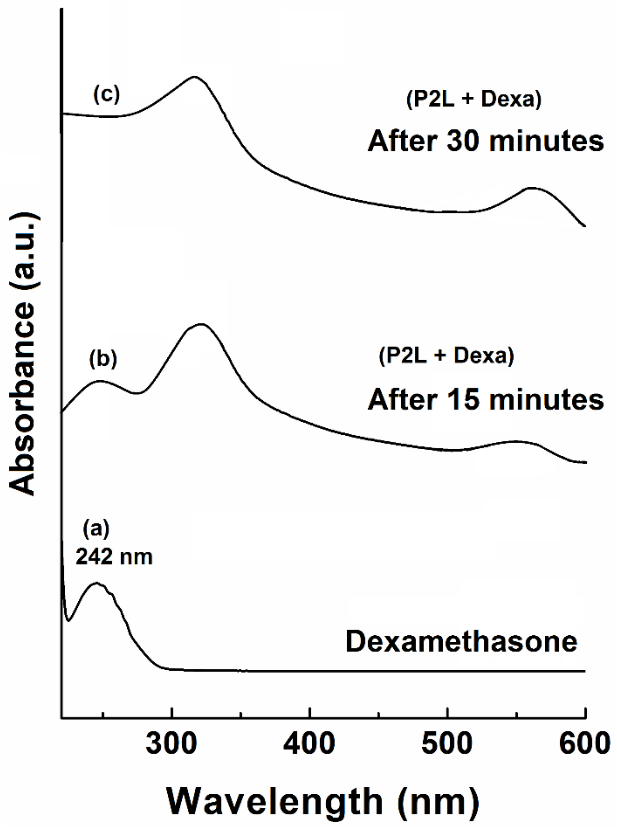

Figure S7. UV-Vis spectra of dexamethasone uptake by P2L.
